# Successful endoscopic management of Bouveret syndrome

**DOI:** 10.1093/jscr/rjac484

**Published:** 2022-10-30

**Authors:** Nyimasata Sanyang, Hiba Shanti, Ameet G Patel

**Affiliations:** Department of General Surgery, King's College Hospital NHS Foundation Trust, Denmark Hill, London, UK; Department of General Surgery, King's College Hospital NHS Foundation Trust, Denmark Hill, London, UK; Department of General Surgery, King's College Hospital NHS Foundation Trust, Denmark Hill, London, UK

## Abstract

We present a frail 83-year-old female with Bouveret syndrome managed using an endoscopic approach. Our patient attended the emergency department with abdominal pain, vomiting and signs of sepsis. She had a recent admission with acute cholecystitis that which had been managed conservatively. Axial imaging revealed aerobilia with a 14 mm common bile duct and a 3.5 cm calculus impacted in the duodenum, in association with a cholecysto-duodenal fistula. After resuscitation, an oesphagoduodenoscopy was performed under general anaesthesia. The large stone was seen impacted in the first part of duodenum. Mechanical lithotripsy and the Kudo snare were employed to fragment the stone and remove large fragments. Bouveret syndrome is rarely managed with success through endoscopy. The syndrome typically occurs in frail, elderly co-morbid patients who would benefit from endoscopic management over open surgery. Despite low success rates historically, endoscopic management is a reasonable and viable option in cases of Bouveret syndrome.

## INTRODUCTION

Severe cholecystitis is common. The rare sequalae is fistulation to surrounding organs, commonly the duodenum resulting in a cholecystoduodenal fistula. This can lead to gall stone ileus (0.3–0.5%) [1]. In 1–3% of these cases, stones are impacted proximally in the stomach or duodenum resulting in gastric outflow obstruction, this is called Bouveret syndrome (BS) [2]. BS was first described by Beaussier in 1770 [3]. In 1896, the French physician Leon Bouveret conferred the eponymous name [3]. This syndrome typically presents in co-morbid females in their seventh to eight decades of life with a female-to-male ratio of 1.4:1 [2].

Patients often present with non-specific symptoms of recurrent vomiting and abdominal pain (70–85%) [4]. Upper gastrointestinal bleeding can be present in 15% of cases [5]. X-ray findings are non-specific and include a dilated stomach and calcified stones which can be seen in 15–20% of cases [3]. Computerized tomography carries the highest sensitivity (93%) for the diagnosis of BS [2]. The combination of aerobilia, gastric outflow obstruction and the presence of a calculus outside of the biliary tree, known as Rigler’s triad, is a diagnostic sign of BS.

BS was traditionally associated with 30% mortality due to extensive co-morbidities. Modern advances in medical, surgical and endoscopic management have improved outcomes; however, mortality rates as high as 12% are still reported [6].

Treatment options include surgical or endoscopic stone retrieval. Endoscopic management carries less risk but also lower success rates. We present a case of successful endoscopic management of BS and discuss surgical and endoscopic management strategies.

## CASE REPORT

**Figure 1:**
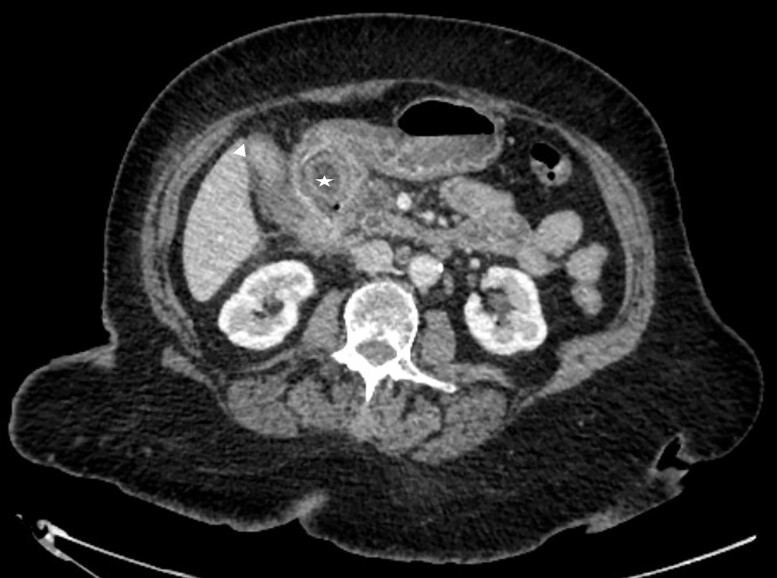
CT scan showing the stone impacted in the first part of duodenum.

**Figure 2:**
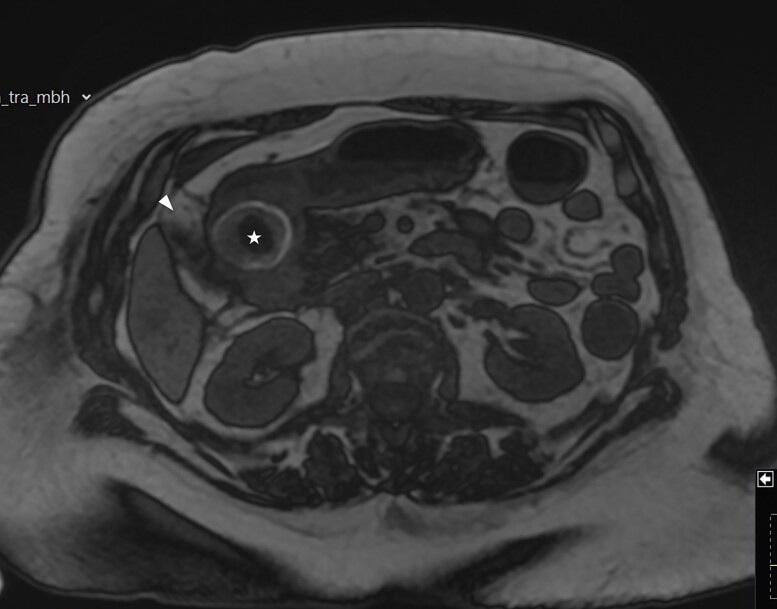
MRI scan showing the gallbladder adherent to the duodenum with an impacted stone in the duodenum.

**Figure 3:**
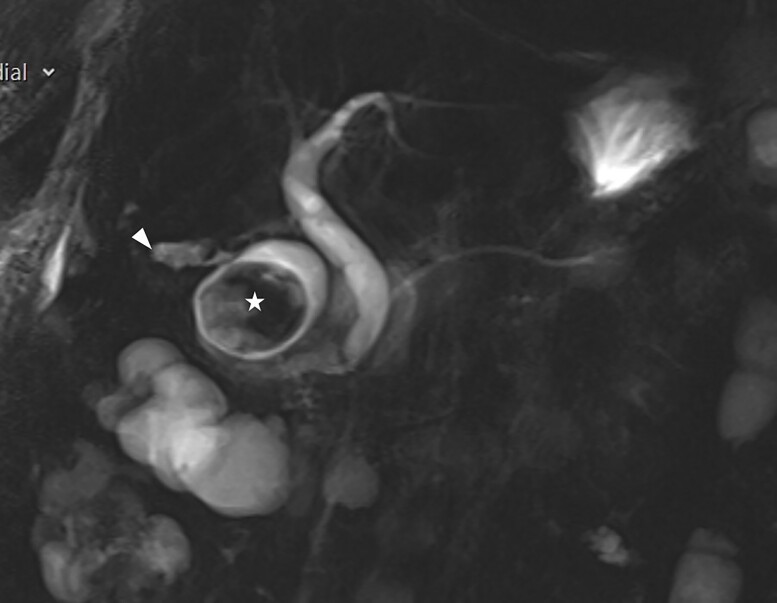
Coronal view of the MRI showing normal biliary tree.

A frail 83-year-old female attended the emergency department with epigastric pain, vomiting and signs of sepsis with a heart rate of 101 and a temperature of 38°C. She had a recent admission with severe cholecystitis that was managed conservatively with intravenous antibiotics. On examination, she had tenderness in the right upper quadrant. Blood revealed an acute kidney injury, a CRP of 189 with normal liver function tests. Axial imaging demonstrated aerobilia with a dilated common bile duct of 14 mm and there was a 3.5 cm stone impacted in the first part of duodenum with evidence of a cholecysto-duodenal fistula (Figs 1–3).

Her medical history included type 2 diabetes mellitus, atrial fibrillation, hypertension, primary hyperparathyroidism, gastro-oesophageal reflux and cauda equina.

After resuscitation, an oesophagogastroduodenoscopy (OGD) was carried out with a view to stone fragmentation and retrieval. A large stone was seen impacted in the first part of the duodenum. Two modalities were used to fragment the stone: mechanical lithotripsy was initially attempted but not successful. The Kudo snare was utilised as a mechanical lithotripter. Large fragments were retrieved using endoscopic nets and baskets to avoid gallstone ileus.

The patient had an uneventful recovery. Her hospital admission was prolonged due to physical deconditioning, and she was discharged 2 weeks later. She has remained well on review in the community.

## DISCUSSION

The first successful endoscopic management of BS using a basket was reported by Dr Guiliana Bedgoni in 1985 [4, 7]. Although endoscopic intervention is attempted in 61% of cases, only 10% result in successful retrieval [2, 8]. A literature review by Dumonceau and Deviere in 2016 reported 61 cases successfully managed by endoscopy in the period from 1978 to 2016 [9]. Successful retrieval is more likely for smaller stones (<4 cm) and stones impacted in the stomach or the first part of the duodenum rather than the distal duodenum [5].

Due to the lower morbidity associated with OGD over surgery, we recommend an endoscopic trial as a first approach. To enhance the success rate, this should be done by an experienced endoscopist. In addition, multiple endoscopic instruments should be utilised simultaneously. These include mechanical lithotripsy, nets and baskets, extracorporeal shock wave lithotripsy, electrohydraulic lithotripsy, laser lithotripsy and duodenal stenting [5]. When solitary methods are employed, mechanical lithotripsy has the highest success rate followed by electrohydraulic lithotripsy [9]. After stone fragmentation, extreme care should be taken to retrieve all large fragments to avoid distal obstruction and gallstone ileus [10–12].

Surgical management is associated with a morbidity of 26% but it is often successful [5]. Laparoscopic surgery is feasible but is less successful if the stones are larger than 5 cm. Gastrotomy or duodenotomy might be required to retrieve stones, whereas surgical resection is rarely indicated.

Synchronous cholecystectomy and management of the fistula remain controversial. It is generally agreed that this should be avoided in the very frail patient [2, 4, 6]. However, it is warranted to extract large stones from the gallbladder, if possible, to avoid recurring obstruction. This has been reported in 10% of patients with non-treated cholecysto-enteric fistula [6].

## DISCLOSURES

All authors declare that there has been no financial support for this work that could have influenced its outcomes.
